# Supplementary material for: CD44v6 Defines a New Population of Circulating Tumor Cells Not Expressing EpCAM
Source: Cancers (Basel). 2021 Oct 2;13(19):4966. doi: 10.3390/cancers13194966 (PMC8508506; doi:10.3390/cancers13194966)
Supplement: Supplementary file 1 [file cancers-13-04966-s001.zip › cancers-1357853-supplementary/Supplementary file S1.pdf]

Pyrosequencing

Biotinylated PCR was performed with an initial denaturation for 15 min at 95°C, followed by 40 cycles of denaturation for 30 s at 94°C, primer annealing for 30 s at 54°C, and extension for 30s at 72°C, followed by a final extension for 10 min at 72°C. All amplification reactions were performed in a DNA Thermal Cycler (Veriti, Thermofisher scientific, France) with Pyromark mastermix containing the Taq polymerase (Qiagen, France).

Mutation	PCR primer sequence	Sense	Sequencing primer
V600	GAC AAC TGT TCA AAC TGA TG	Up	AGTAAAAATAGGTGATT
	Biotin-AGTAAAAATAGGTGATT	Down	

Primer sequences for the amplicons used for PCR amplification and sequencing analysis are shown in the table above.

DNA product consisted of amplified tumoral DNA from control cells. Templates for the pyrosequencing analysis were used as recommended by the manufacturer. Real-time pyrosequencing was performed at 28°C in an automated, PyromarkQ24. Prior to analysis, the enzymes and each of the four dNTPs (PyroMark Q24 Gold Reagents, Qiagen, France) were loaded into a special cartridge that was mounted in the PyromarkQ24 instrument.

ddPCR

The presence of the *KRAS* G12V mutation was assessed using the QX200 ddPCR System (Bio-Rad, Marnes-la-Coquette, France). Forward and reverse gene-specific primers and fluorescent hydrolysis probes specific for either the mutant (FAM, dHsaCP2500592) or wild-type (HEX, dHsaCP2000006) were obtained from Bio-Rad. Briefly, DNA extracted from FFPE tumor sample and CTC sample from the same patient was used in a ddPCR reaction with ddPCR supermix (Bio-Rad). The reactions were then emulsified using a QX200 AutoDG droplet generator, transferred to 96-well plates and amplified using the following cycling conditions: 95°C for 10 min; 40 cycles of 94°C for 30 sec, and 55°C for 1 min; and 98°C for 10 min. After amplification, plates were read and fluorescence signal of individual sample droplets were analyzed with a QX200 droplet reader. The mutant allele frequency, relative to that of wild-type, was determined using QuantaSoft V.1.7.4 software, by applying a correction, based on the Poisson distribution, to the number of droplets positive for either mutant or wild-type DNA. In each experiment, a DNA reference standard control sample that harbored a *KRAS*

G12V mutation at a frequency of 5% and a DNA reference standard control sample that without the *KRAS* G12V mutation were used to check the sensitivity and specificity of our techniques. Water was also added instead of DNA to evaluate the absence of contamination.

NGS analysis

Libraries were prepared as previously described as well as the further sequencing and analysis [1].

1. Vendrell, J. A., Quantin, X., Serre, I. & Solassol, J. Combination of tissue and liquid biopsy molecular profiling to detect transformation to small cell lung carcinoma during osimertinib treatment. *Ther. Adv. Med. Oncol.* **12**, (2020).
