# Supplementary figures and images for: CD44v6 Defines a New Population of Circulating Tumor Cells Not Expressing EpCAM
Source: Cancers (Basel). 2021 Oct 2;13(19):4966. doi: 10.3390/cancers13194966 (PMC8508506; doi:10.3390/cancers13194966)

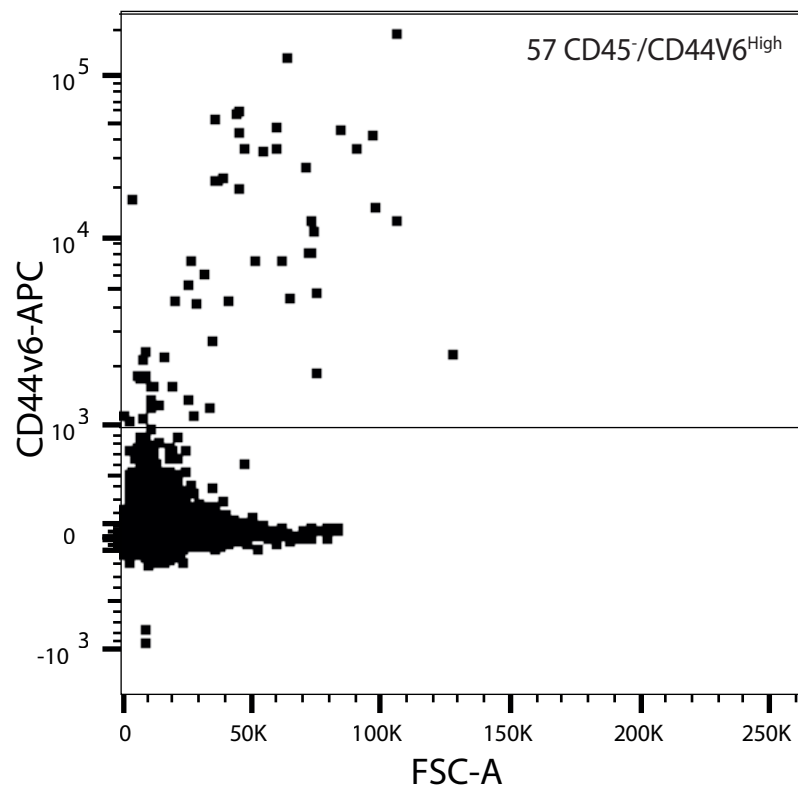

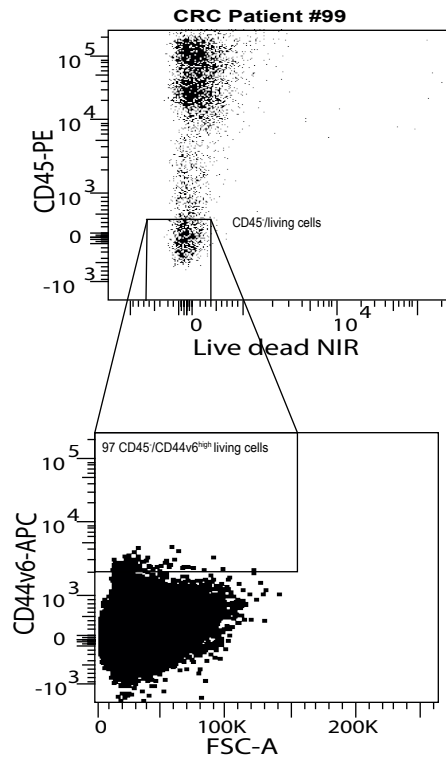

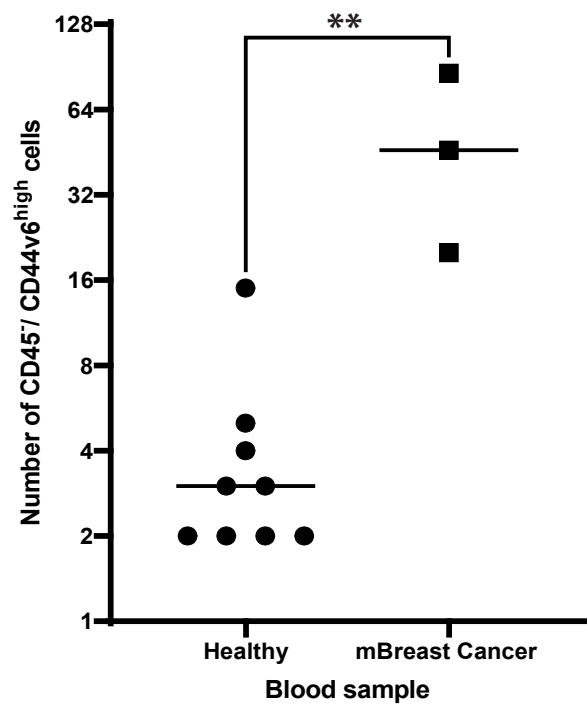

Supplement: Supplementary file 1 [file cancers-13-04966-s001.zip › cancers-1357853-supplementary/cancers-1357853-suppl-resubmit.pdf]
